# Geospatial datasets describing route geometry and ultrafine particulate matter dosage for children during shortest-distance and lowest-dosage school commutes in Toronto, Canada

**DOI:** 10.1016/j.dib.2019.104792

**Published:** 2019-11-11

**Authors:** Spencer Elford, Matthew D. Adams

**Affiliations:** University of Toronto Mississauga, Canada

**Keywords:** Ultrafine particulate matter, Air pollution exposure, Dosage, Schools, Active transportation, Modelling

## Abstract

The data in this article provides route geometries and ultrafine particulate dosage information for a simulation of the home-to-school walking commute for children at 296,862 residential addresses in the city of Toronto, Canada. The datasets include dosage estimates that use a modelling approach that accounts for terrain, physiology and spatial variability in ambient UFP concentrations. The dataset provides simulated routes that describe both the shortest distance route, as well as the lowest UFP dosage route. Dosage and route information are provided in both polyline (route) and point (origin address) feature classes. Included in this article is a brief description of the simulation approach taken to generate the data. For discussion and complete description of the modelling approach, please refer to “Exposure to ultrafine particulate air pollution in the school commute: Examining low-dose route optimization with terrain-enforced dosage modelling” [1].

**Table:** Specifications Table

Subject area	*Atmospheric Science*
More specific subject area	*Air Pollution Exposure*
Type of data	*Vector Spatial Data (ESRI Geodatabase & Feature Class)*
How data was acquired	*Simulation of routes generated in ArcGIS 10.5 using the network analyst extension. Modelling of ventilation rate, walking speed, energy expenditure and dosage conducted in R (included R script).*
Data format	*ESRI Feature Datasets in a File Geodatabase (raw)*
Experimental factors	*A network dataset was built to allow only travel on pedestrian-supported links. Highways, railways, and transit ways are restricted. Similarly, travel routes are only supported within school-board defined enrolment boundaries, which are included as barriers in the network. Several spatial datasets are used in generating this data, and include: Land-use related Datasets, Address repositories, High Resolution Digital Elevation Models, Pedestrian Network Datasets, and School Enrolment Boundaries.*
Experimental features	*Network Analysis in ArcGIS 10.5 is used to obtain both the shortest distance route and the lowest Ultrafine particulate (UFP) dosage route for 296,862 home locations to their designated school. Routes are divided into 10 m segments where models for air pollution concentration, energy expenditure, walking speed, and ventilation rate are used to obtain estimated dosage of UFP. Segment attributes are aggregated for each complete route and presented as a single trip dosage.*
Data source location	*Toronto, Ontario, Canada*
Data accessibility	*The data are available within this article.*
Related research article	*Exposure to ultrafine particulate air pollution in the school commute: examining low-dose route optimization with terrain-enforced dosage modelling* [1] *Elford S, Adams M.D.* *Environmental Research, Volume 178, November 2019, 108,674*

**Table:** 

**Value of the Data** • The data describes the route geometry and dosage of ultrafine particulate matter (UFP) for the home-to-school commutes of all 296,862 residential addresses found to be within walking distance of their designated school in the city of Toronto. The information is presented as both polyline and point datasets to support usage in spatial analysis applications for exploring geographic trends in air pollution exposure. • The data provides geometry and dosage information for both shortest-distance routes and lowest-dosage routes, offering insight for researchers on how changes in commute patterns may affect dosage for some locations in Toronto. • The data demonstrates the applicability of dosage models that incorporate environmental factors (e.g. slope, ambient UFP concentration) and physiology through a workflow that is both flexible and scalable, allowing researchers to apply them to varied populations and locations where data is present. • The data may be applied to broad-scale examination of school-commute related dosage, and the factors that may be related to, or impacted by exposure (e.g. socioeconomic status, urban morphology/built environment, zoning and land-use planning). The dataset contains a sufficiently large sample size and coverage to support further analysis.

## 1 Data

The data is provided as an ESRI File geodatabase containing 3 feature classes. Two feature classes are polylines that present route geometries and ultrafine particulate matter dosage attributes for the walk to school commute from 296,862 residential addresses to their assigned school in the city of Toronto. The first feature class describes routes calculated that represent the shortest distance (“Routes_ShortestDistance”). A second feature class describes routes that are expected to incur the lowest UFP dosage (“Routes_LowestDosage”). Both feature classes share the same fields, providing a school ID (“FacilityID”), Home ID (“IncidentID”), UFP dosage estimate in pUFP (“Dose”), and the length of the commute (“Shape_Length”). The third feature class (“Homes_RouteDosageSummary”) contains point locations for all residential origin locations with joined attributes that facilitate comparison of shortest-distance and lowest-dosage routing solutions. Fields included are; unique school identifier (“FacilityID”), unique home identifier (“IncidentID”), UFP dosage for both route solutions (“Dose_Shortest” and “Dose_LowestDose”), change in dosage (“Dose_Reduction”), route distance for both route solutions (“Length_Shortest” and “Length_LowestDose”), change in walking distance (“Length_Change”), and fields describing percentage reduction in dosage (“Percent_DoseReduction”), and percentage increase in length (“Percent_LengthChange”) for the low-dosage route solution.

Included as supplementary information with this article is an R script that provides the modelling functions used to obtain terrain-enforced walking speed, energy-expenditure, ventilation rate, and dosage. This script (TerrainEnforcedCommuteDosage.R”) assumes environmental attributes for slope, elevation and ambient pollution concentration are pre-obtained.

## 2 Experimental design, materials, and methods

Routes were simulated using residential addresses as origin points and school locations as the destination point. In ArcGIS 10.5, the network analyst extension was used to generate routes for 296,862 residential address in the Toronto municipal area. We imposed a maximum travel distance limit set at 1600-m, which corresponded to the school-bus access cut-off. Additionally, residential addresses directly adjacent to their school's property line were also excluded, as the route solver did not allow travel perpendicular to the network link (i.e crossing the road). Finally, travel was only permitted within each school's designated enrolment boundary, and no-cross school boundary commutes were permitted. To enforce this, enrolment boundaries were considered restriction barriers in the network solver. A network dataset is built from the city of Toronto roads dataset and is supplemented with trails and sidewalk data from OpenStreetMap and the Toronto sidewalk inventory. The network is modified to only accommodate pedestrian travel. This is achieved by removing vehicle-only network segments (i.e highways, transitways, rail lines). We used distinct routing solutions that simulate commutes under two scenarios, the lowest travel distance route, and the lowest UFP dosage route.

In the first scenario, distance acted as the cost attribute and the result was the shortest possible travel path to school. All routes were generated and split into equal length 10-m segments. Start and end point vertices were extracted for every segment and directional slope was obtained from extracted elevation values on a 1-m resolution digital elevation model. Dosage was calculated for each segment making up a route using several models that estimate energy expenditure [2], walking speed [3], and ventilation rate [4]. The models account for the effects of physiology and terrain on estimating ventilation rate and travel velocity. A land-use regression model was used to obtain estimates of mean annual ambient UFP concentrations at the midpoint of every 10-m segment [5]. We used a respiratory dosage equation to estimate UFP route dosage (1).

(1) $$D_{r}=\overset{n}{\underset{i=1}{\sum }}\frac{10}{V_{i}}*{V_{e}}_{i}*C_{i}$$

where *D*_*r*_ is respiratory dosage for a route *r* as a count of UFP particulates (pUFP). Route dosage is the sum of dose for all 10-m links, where *i* = 1 represents the first link out of *n* links that make a route, *V*_*i*_ is the terrain-enforced walking speed for link *i* in meters per second. *V*_*e*_ is the terrain-enforced ventilation rate for link *i*, in cubic meters per second. *C*_*i*_ is the modelled ambient concentration of UFP, in units of pUFP per cubic meter. A full description of the modelling approach is provided in a co-submitted article (Elford and Adams, 2019 [1]). Variables for elevation, length, and slope were calculated in ArcGIS 10.5, and walking speed, ventilation rate, energy expenditure, and dosage were computed in R using the script included with this article (TerrainEnforcedCommuteDosage.R).

Simulation of the lowest UFP dosage scenario required modification to the above method. The network dataset was split into 10m-links and each is given 2 dosage cost estimates based on direction of travel (to account for differences in dosage between uphill and downhill travel). Dosage was used as a cost attribute instead of distance and was accumulated during traversal over a network link. The 1600-m distance limit was removed while enrolment boundary restrictions were still maintained. Using the network solver, routes are generated for all homes used in scenario 1, and dosage is re-evaluated in R.

Route attributes for both the shortest distance and lowest dosage routes are joined to a point feature class of address locations (Residential Origin points) using a unique identifier (“IncidentID”). Dosage reduction potential and distance requirements necessary to acquire a lower dosage are then calculated presented in the point feature dataset.

## References

[1] Elford S., Adams M.D. (2019). Exposure to ultrafine particulate air pollution in the school commute: Examining low-dose route optimization with terrain-enforced dosage modelling. Environ. Res..

[2] Kramer P.A. (2010). The effect on energy expenditure of walking. Am. J. Hum. Biol..

[3] Tobler W. (1993). Three Presentations on Geographical Analysis and Modeling: Non-isotropic Geographic Modeling Speculations on the Geometry of Geography Global Spatial Analysis, Technical Report for the National Center for Geographic Information and Analysis.

[4] U.S. Environmental Protection Agency (EPA) (2009). Metabolically Derived Human Ventilation Rates : A Revised Approach Based upon Oxygen Consumption Rates.

[5] Weichenthal S., Van Ryswyk K., Goldstein A., Shekarrizfard M., Hatzopoulou M. (2016). Characterizing the spatial distribution of ambient ultrafine particles in Toronto, Canada: a land use regression model. Environ. Pollut..

